# SIMSISH Technique Does Not Alter the Apparent Isotopic Composition of Bacterial Cells

**DOI:** 10.1371/journal.pone.0077522

**Published:** 2013-10-29

**Authors:** Olivier Chapleur, Ting-Di Wu, Jean-Luc Guerquin-Kern, Laurent Mazéas, Théodore Bouchez

**Affiliations:** 1 UR HBAN, Irstea, Antony, France; 2 U.759, INSERM, Orsay, France; 3 Laboratoire de Microscopie Ionique, Institut Curie, Orsay, France; University of Vienna, Austria

## Abstract

In order to identify the function of uncultured microorganisms in their environment, the SIMSISH method, combining *in situ* hybridization (ISH) and nanoscale secondary ion mass spectrometry (nanoSIMS) imaging, has been proposed to determine the quantitative uptake of specific labelled substrates by uncultured microbes at the single cell level. This technique requires the hybridization of rRNA targeted halogenated DNA probes on fixed and permeabilized microorganisms. Exogenous atoms are introduced into cells and endogenous atoms removed during the experimental procedures. Consequently differences between the original and the apparent isotopic composition of cells may occur. In the present study, the influence of the experimental procedures of SIMSISH on the isotopic composition of carbon in *E. coli* cells was evaluated with nanoSIMS and compared to elemental analyser-isotopic ratio mass spectrometer (EA-IRMS) measurements. Our results show that fixation and hybridization have a very limited, reproducible and homogeneous influence on the isotopic composition of cells. Thereby, the SIMSISH procedure minimizes the contamination of the sample by exogenous atoms, thus providing a means to detect the phylogenetic identity and to measure precisely the carbon isotopic composition at the single cell level. This technique was successfully applied to a complex sample with double bromine – iodine labelling targeting a large group of *bacteria* and a specific *archaea* to evaluate their specific ^13^C uptake during labelled methanol anaerobic degradation.

## Introduction

Over the past decades, molecular biology approaches have allowed to overcome limitations associated to cultivation-dependent methods, which were greatly underestimating actual diversity [1], and enabled strong advances in microbial communities’ quantification and classification. However, most molecular-based technologies are still unable to directly link phylogeny and biochemical processes. Consequently, the *in situ* ecophysiology of many key microorganisms still remains poorly documented. Linking phylogenetic information to function in complex environmental communities is thus one of the main challenges of microbial ecology.

In this context, different methods coupling isotope-labelling experiments and *in situ* hybridization have been developed to investigate the ecophysiology of microbial populations. They reveal the specific uptake of isotopically labelled substrates within individual cells and the phylogenetic affiliation of the very same cells. Among them, MarFISH [2]–[4] combines fluorescence *in situ* hybridization (FISH) and microautoradiography (mar) but is limited to radioactive isotopes. Raman FISH [5] allows the detection of isotopes incorporated into cells using Raman microscopy. More recently, new methodologies involving high spatial resolution secondary ion mass spectrometry (nanoSIMS) have been developed [6]–[9]. They allow more precise isotopic measurements and a better spatial resolution than previous techniques. Although these methods all rely on the combination of rRNA-based *in situ* hybridization with stable isotope imaging based on nanoSIMS, they differ notably by the hybridization procedure. Secondary Ion Mass Spectroscopy *In situ* Hybridization, SIMSISH, is based on the direct hybridization of halogenated DNA probes [6] whereas ELFISH or HISHSIMS (respectively Element Labeling-Fluorescent *In situ* Hybridization and Halogen *In situ* Hybridization Secondary Ion Mass Spectroscopy) uses catalyzed reporter deposition fluorescence *in situ* hybridization [10] with horseradish-peroxidase-labeled oligonucleotide probes and halogen-containing tyramides for the identification of microorganisms [7]–[9]. These methodological differences could imply different consequences on the measurement of isotopic enrichment in cells. Indeed, treatment and preparation of samples prior to nanoSIMS analysis (e.g. chemical fixation of samples, or the application of genetic probes during hybridization) are factors that can modify the initial composition of the microbial cells [11]. No detailed descriptions of these effects are available up to date.

Yet the accuracy of the absolute value of isotopic enrichment measurement together with a specific phylogenetic identification is very important to have precise information on microorganisms’ functions in complex environments or to establish functional models. In the present study, the option to measure isotopic ratios in probe-labelled single cells with a nanoSIMS was evaluated using SIMSISH technique [6]. This technique requires introducing halogens (or any other element rarely present in biomass) into cells via halogenated 16S rRNA-targeted probes, after isotopic enrichment of cells with carbon (^13^C) or any element of biological interest. Hybridization is performed on PFA-fixed and ethanol-permeabilized cells. Oligonucleotide probe-conferred hybridization signal (halogen) and isotopic enrichment are measured directly in microbial cells with the nanoSIMS. Even if only a limited number of probes and exogenous atoms are introduced (and endogenous atoms removed) into cells during the fixation and hybridization procedures, the samples might be modified resulting in differences between the original isotopic content and the apparent isotopic composition of cells measured by nanoSIMS [6], [11]. The present study was designed to evaluate with precision the influence of these procedures on the quantification of cells’ stable isotopic composition focusing on carbon. Single cell level analyses, realized with nanoSIMS, were compared to reference analyses, at population level on cells pellets, realized with elemental analyser-isotopic ratio mass spectrometer (EA-IRMS). Analyses were performed on a set of *E. coli* cells isotopically labelled at different levels of ^13^C enrichment. The possibility of simultaneously identifying and measuring the isotopic enrichment of several microorganisms in one experiment was also evaluated with a double labelling experiment.

## Materials and Methods

Unless specifically mentioned, all experimental procedures were performed according to [6].

### 1. Bacterial Strain and Growth Conditions

Pure cultures of *Escherichia coli* JM109 were grown in a defined medium containing per litre of distilled water, 1 g of NH_4_Cl, 0.2 g of MgSO_4_·7 H_2_O, 6 g of NaH_2_PO_4_·H_2_O, 3 g of K_2_HPO_4_, 0.5 g of NaCl and 0.01 g of CaCl_2_, supplemented with 10 mg.ml^−1^ glucose as the sole organic carbon source [6]. For ^13^C labelling, unlabelled glucose was substituted by ^13^C_6_-glucose (99 atom % at ^13^C, Cambridge isotopic laboratory, UK). 10 groups of cells were prepared from minimal culture media with 10 different isotopic compositions of ^13^C (1.10%, 2.08%, 3.06%, 6.00%, 10.89%, 20.68%, 40.26%, 59.84%, 79.42% and 99.00% of ^13^C in glucose, measured with EA-IRMS). Strains were cultivated aerobically at 37°C and harvested in stationary phase after 20 h of incubation. For each isotopic composition, grown cell culture was divided in three batches. Cells pellets were washed once with 1× phosphate-buffered saline (PBS, Sigma). One was kept as “untreated cells”, the other two were used subsequently for fixation and hybridization procedures.

### 2. Complex Sample

A complex sample was recovered from an anaerobic batch digester (120 ml) inoculated with municipal solid waste landfill leachate and fed with ^13^C-labeled methanol (99% in ^13^, Cambridge isotopic laboratory, UK, concentration of 4.75 g/l) after 25 days of incubation. Specific degradation parameters and microbial analysis are described in [12].

### 3. Fixation and Hybridization Procedures

For fixation, cells pellets were re-suspended in 200 µl of 1×PBS (Sigma) and 600 µl 4% paraformaldehyde (Sigma) as fixative. After 3 h of incubation at 4°C, tubes were centrifuged (11 000 g, 10 min) and pellets were washed once again with 1×PBS and re-suspended in 500 µl of 1×PBS and 500 µl of pure ethanol. Fixed cells were stored at −20°C.

For *in situ* hybridization, 10–100 µl of fixed cells were washed in 400 µl of hybridization buffer (0.9 M sodium chloride, 20 mM Tris-HCl, 0.1% SDS and 20% of formamide). The resulting cell suspension was subjected to vortex (1 min) and cells were recovered by centrifugation and re-suspended in 20 µl of pre-heated hybridization buffer. Two microlitres of probe (50 ng.µl^−1^) were added and the suspension was incubated during 2 h at 46°C. Cells were then recovered by centrifugation (11 000 g, 10 min) and washed for 15 min in wash buffer (0.215 M sodium chloride, 20 mM Tris-HCl, 5 mM EDTA, 0.1% SDS) at 48°C. Finally, the cells were centrifuged for 10 min at 11 000 g and re-suspended in 50 µl of sterile ice-cold ultrapure water. Hybridization was performed with a generalist bacterial iodinated EUB338 probe I_6_-Eub338-Cy3 (5′-Cy3-GcTGccTcccGTAGGAGT-3′ c = 5-iodo-2′deoxycytidine, synthesized by Proligo) [6]. Quality of hybridization was checked before subsequent analysis. Quality of fluorescent signal was checked with laser confocal microscope (Figure S1). An intense and bright signal was always obtained. Quality of halogen signal was checked with nanoSIMS (Figure S2). At least 85% of cells exhibited a clear halogen labelling.

The same protocol was applied to a complex sample. Two probes were used: generalist bacterial brominated EUB338 probe Br_8_-Eub338-Cy3 (5′-Cy3-GcTGccTcccGTaGGaGT-3′ a = 5-bromo-2′deoxyadenosine c = 5-bromo-2′deoxycytidine, synthesized by Proligo) and specific archaeal iodinated MS1414 probe (targeting *Methanosarcina* genera) I_9_-MS1414-Cy3 (5′-Cy3-cTcAcccATAccTcATcGGG-3′ c = 5-iodo-2′deoxycytidine, synthesized by Proligo).

### 4. ^13^C Abundance Measurement with EA-IRMS

Pellets of untreated (non-fixed and non-hybridized), fixed and fixed/hybridized *E. coli* cells were dried overnight (55°C) and subjected to isotopic analysis (^13^C/^12^C) by EA-IRMS from Thermo Electron (Germany). For each pellet, two samples of about 150 µg each were transferred to ultrapure tin container (Thermo) and analyzed, except for fixed/hybridized cells (only one sample).

### 5. ^13^C Abundance Measurement with nanoSIMS

#### 5.1. E. coli pure culture

In order to provide a more reliable and comparable measurement of the difference in ^13^C content, untreated and fixed/hybridized *E. coli* cells of the same isotopic composition were mixed and observed together. Hybridized cells were distinguished from untreated cells by the iodine map. Fixed cells were not analysed as they could not be distinguished from untreated cells with nanoSIMS observation.

1 µl of fixed/hybridized sample was mixed with 1 µl of untreated cells (1∶50 dilution of pure culture pellets). Drops of the mixtures were spread on 7 mm×7 mm high-purity silicon chips (Silicon Quest International) cleaned with ultrapure water and absolute ethanol. After drying in a oven at 55°C overnight, samples were imaged with an epifluorescent microscope to localise hybridized cells on the chips for subsequent NanoSIMS measurements.

Silicon chips were then introduced into a NanoSIMS-50 instrument (CAMECA, Gennevilliers, France) equipped with caesium ion source. For the present study, by using a Cs+ primary ion beam tightly focused to a typical probe size of about 100–150 nm in diameter, up to five of the secondary ion species (among 12C–, 13C–, 12C14N–, 13C14N–, 32S–, 127I–) were targeted [6].

The area of interest was first selected by rapid survey with detection of iodine signal and 32S– signal in order to localize hybridized cells in the whole biomass. Precise abundance determination required a second acquisition with different detectors settings. After careful Cs+ ion implantation to get steady state emission of C^−^, the acquisition of ^13^C^−^ and 12C- images was carried out using multiframe mode. The typical raster size was 30 µm with an image definition of 512×512 pixels and the Dwell time was 500 µs per pixel. At least 15 frames were acquired but only the sum of the 10 last frames with best intensity stability were used for ^13^C and ^12^C ratio determination so as to reduce the possible mass fractionation due to variation in ion emission for the two isotopic ions. Proper alignment between each frame was ensured using ImageJ software (Wayne Rasband, http://imagej.nih.gov/ij).

In NanoSIMS microprobe, ^13^C^−^ to ^12^C^−^ ions are recorded simultaneously in two different detectors (electron multipliers, ion counting mode). The response of these two detectors has to be well balanced to allow precise measurement of the ratio between ^13^C^−^ and ^12^C^−^ ions. Furthermore, to ensure the accuracy of the ^13^C abundance determination, the obtained ratio has to be corrected by the mass fractionation mainly induced by the ion emission process. For these reasons, a reference material with known ^13^C content was employed to calibrate the ratio measured by the 2 detectors.

A calibration constant (K) was established based on the analysis of such reference material (r).
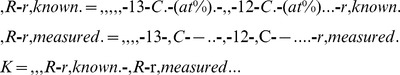



Then, for each ^13^C^−^ to ^12^C^−^ ratio measured from an unknown sample (s) under the same operating conditions, the ^13^C content was determined by using the above calibration constant.
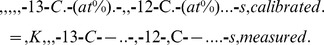



For the present study, we used a homogeneous Spurr resin at natural abundance (BioValley) as reference material. This Spurr resin has a nominal composition of 34.8% of C, 59.6% of H, 5.6% of O and 0.06% of N which is quite similar to biological sample. It was first calibrated for its C composition by EA-IRMS technique against a certified reference material (USGS-24, graphite). The calibrated 13C (at%) content is 1.079335±0.000108 (0.01% relative, 1SD). Thin sections from the same block of resin were placed onto clean Si chip to be analyzed by NanoSIMS to provide the calibration constant (K). One thin resin section was measured with the same operating conditions during each measurement session on *E. Coli* samples as a control. During the present study, the measured 13C (at%) was 1.0562±0.00506 (0.48% relative, 1SD) for 4 measurement sessions and no significant change was observed. Therefore, a unique calibration constant (K) of 1.0222 was applied to all the measurements. Such calibration procedure was applied directly to the acquired images based on a pixel-to-pixel calculation.

Using ImageJ software and the obtained ^13^C and ^12^C images, the ^13^C and ^12^C ratio for each pixel was calculated to generate a ^13^C content map.

At least two different areas were analysed for each sample.

#### 5.2. Complex sample

The same protocol was used for complex sample. 1 µl of fixed/hybridized sample was spread on a silicon chip. Br^−^ ion was also targeted and used to localize hybridized cells in the total biomass.

### 6. Student’s t-Test

Paired difference Student’s t-Tests were performed with R on isotopic ratio measurements to estimate the influence of fixation and hybridization. Tests were performed on normalized data, which means that isotopic enrichment value of each group of cells (untreated, fixed and fixed/hybridized) was divided by the corresponding enrichment of untreated cells.

## Results and Discussion

### 1. Influence of SIMSISH Procedure on Isotopic Composition of *E. coli* Cells

Cells of the ten different groups of pure cultures were fixed, permeabilized and hybridized. Their isotopic composition (^13^C) was measured with both EA-IRMS and nanoSIMS. To evaluate the degree of modification in isotopic composition, the ^13^C isotopic abundance obtained after hybridization was compared to the ^13^C isotopic abundance in untreated cells (sampled directly from the culture, recovered by centrifugation and dried) and in fixed and permeabilized cells (not hybridized, EA-IRMS comparison only).

#### 1.1. EA-IRMS measurements

**Figure 1:**
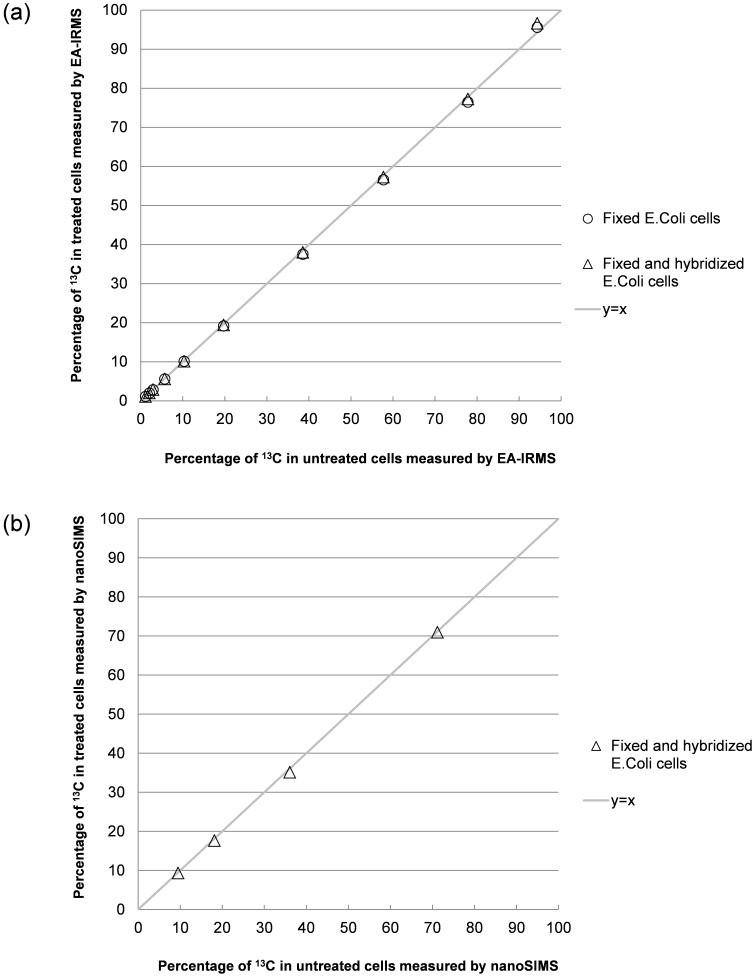
Isotopic composition of untreated, fixed and fixed hybridized cells measured with EA-IRMS and nanoSIMS. (a) ^13^C isotopic composition of fixed, and fixed/hybridized E. coli cells measured with EA-IRMS compared to ^13^C isotopic composition of untreated cells – (b) ^13^C isotopic composition of fixed/hybridized E. coli cells measured with nanoSIMS compared to ^13^C isotopic composition of untreated cells.

The labelling of *E. coli* with 13C was measured on cells’ pellets with EA-IRMS for untreated, fixed and fixed/hybridized cells. Table 1 summarizes the results obtained (detailed values are presented in table A in File S1). Isotopic enrichment of untreated cells is slightly inferior to isotopic enrichment of culture media, probably because cells consumed preferably unlabeled glucose. The isotopic values are nearly identical for untreated cells, fixed cells and fixed/hybridized cells, which means that the procedures used for SIMSISH have a very limited influence on the apparent isotopic composition of microbes measured with EA-IRMS at population level. This is confirmed by figure 1(a) that shows the isotopic enrichment distribution of fixed cells and fixed/hybridized cells as functions of isotopic enrichment of untreated cells. Both distributions are linear, with a slope very close to 1. More precisely the similarity of the measurement for untreated and fixed, and untreated and fixed/hybridized cells was tested with paired difference Student’s t-Test. Data were not significantly different (p<0.06), showing that isotopic measurements performed on fixed or fixed/hybridized cells pellets are statistically indistinguishable from those obtained on untreated cell pellets.

**Table 1:** Isotopic composition of E. coli cells measured with EA-IRMS.

Sample	Percentage of ^13^C in culture media									
	1.10%	2.08%	3.06%	6.00%	10.89%	20.68%	40.26%	59.84%	79.42%	99.00%
(a) Untreated *E. coli* cells	1.11% (0.01)	2.01% (0.01)	2.91% (0.01)	5.69% (0.01)	10.31% (0.01)	19.71% (0.02)	38.53% (0.02)	57.7% (0.00)	77.82% (0.05)	94.29% (4.50)
(b) Fixed *E. coli* cells	1.1% (0.00)	1.96% (0.02)	2.84% (0.05)	5.58% (0.06)	10.11% (0.09)	19.19% (0.12)	37.53% (0.29)	56.57% (0.13)	76.46% (0.26)	95.58% (0.60)
(c) Fixed and hybridized *E. coli* cells	1.10% -	1.99% -	2.88% -	5.59% -	10.11% -	19.39% -	38.01% -	57.24% -	77.24% -	96.63% -

Isotopic composition of untreated, fixed, and fixed/hybridized *E. coli* cells measured with EA-IRMS on dried cells pellets. Standard deviation is given in parentheses.

#### 1.2. nanoSIMS measurements

**Figure 2:**
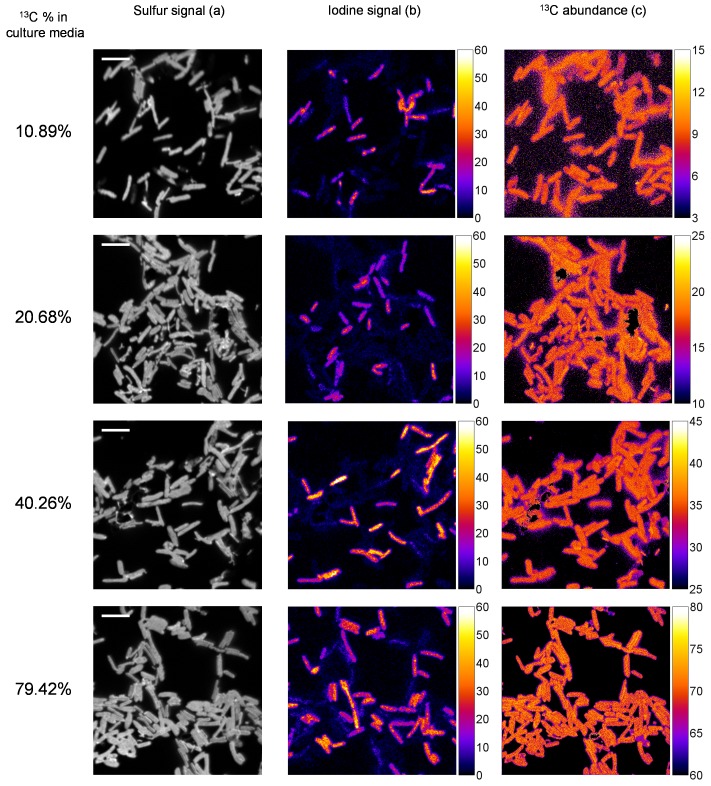
NanoSIMS images obtained for mixed untreated and fixed/hybridized cells grown in enriched culture media. NanoSIMS images obtained for mixed untreated and fixed/hybridized cells grown in enriched culture media with nominal ^13^C abundance of 10.9, 20.7, 40.3 and 79.4%. Panel (a) shows the secondary ion of ^32^S^−^ image as an image of total biomass (scale bar : 5 µm). Panel (b) shows the secondary ion of ^127^I^−^ image as an indication of hybridized cells. (c) ^13^C Isotopic abundance map of corresponding area.

*E. coli* cells grown in 10.9, 20.7, 40.3 and 79.4% of ^13^C were chosen to be observed with nanoSIMS, at single cell level. In a first step, the lateral resolution of nanoSIMS probe was evaluated on a mixture of these cells (Figure S3). NanoSIMS provides an efficient discrimination between lateral neighbour cells. In a second step, untreated and fixed/hybridized cells of each group were analysed simultaneously. A set of images showing sulfur and iodine emission as well as corresponding ^13^C abundance map is presented in figure 2.

Sulfur is an indication of total biomass. Iodine signal enables an identification of hybridized cells from the non-hybridized (untreated) ones (Figure 2(a)). Each cell has rather homogeneous 13C distribution and the 13C content is very close for cells within each image and group (Figure 2(b)). The average ^13^C abundance and standard deviation were calculated for each type of cells observed (between 30 and 60 cells in each group of cells). Table 2 summarizes the results obtained (detailed values are presented in tables S2 and S3 in File S1).

**Table 2:** Mean isotopic composition of individual cells measured with nanoSIMS.

Sample	Percentage of ^13^C in culture media			
	10.89%	20.68%	40.26%	79.42%
(a) Untreated *E. coli* cells	9.47% (0.24)	18.12% (0.36)	36.06% (0.55)	71.16% (0.95)
(b) Fixed and hybridized *E. coli* cells	9.35% (0.18)	17.67% (0.23)	35.16% (0.32)	70.98% (0.93)

Mean isotopic composition of untreated and fixed/hybridized *E. coli* cells measured with nanoSIMS on individual cells. Standard deviation is given in parentheses.

Standard deviations are very low within one type of cells, regardless of the area of the sample observed (not shown) or fixation/hybridization experiment. First of all, it means that the untreated cells used for the experiment presented a homogeneous enrichment. It also signifies that the procedures of SIMSISH have a very repeatable and homogeneous effect on all cells isotopic ratio measured with nanoSIMS at the single cell level.

Moreover, as already observed in EA-IRMS measurements, ^13^C abundance obtained with NanoSIMS measurements for fixed/hybridized cells differs only slightly from ^13^C abundance obtained for untreated cells, which means that the procedures used for SIMSISH have also a very limited influence on the apparent composition of microbes at single cell level. Figure 1(b) shows that isotopic enrichment distributions of fixed/hybridized individual cells is a linear function of isotopic enrichment of untreated individual cells with a slope very close to 1. Paired difference Student’s t-Test shows that data are not significantly different (p<0.06). It means that isotopic measurements performed on fixed and hybridized individual cells are statistically indistinguishable from those obtained on untreated individual cells.

#### 1.3. EA-IRMS and nanoSIMS comparison

The isotopic compositions determined independently at single-cell level, from the NanoSIMS observation, and at population level, with EA-IRMS, are in overall agreement for each cell culture (Table 1 and 2). However, the 13C abundance measured by nanoSIMS is slightly lower than the one by EA-IRMS, even if a quasi-linear relation can be established between these measurements. Several hypotheses could explain this observation. In this work, the calibration of nanoSIMS was realized with a homogeneous resin at natural isotopic carbon composition. This reference value is at the low end of the scale to be measured. Therefore, a cross calibration with a 13C enriched resin would be useful to check if the NanoSIMS detection system has linear response from low 13C content up to high 13C content. In addition, extra mass fractionation may occur as a result of difference in ion yield emission between cells and resin. A standard consisting of a matrix similar to cells samples could help to reduce uncertainty in measurement of isotopic composition, but is not yet identified [13]. In the absence of such a standard, an internal calibration with cells of known isotopic composition could be used. Further studies are needed to provide nanoSIMS with a homogeneous and calibrated reference.

In summary, EA-IRMS and nanoSIMS measurements show independently that the fixation and hybridization procedures used in SIMSISH technique have little influence on the carbon isotopic composition of *E. coli* treated-cells. This indicates that only a very small amount of exogenous carbon is introduced in cells during SIMSISH procedure(a theoretical calculation presented in supporting information suggests that the amount of carbon introduced with probes during SIMSISH procedure is less than 0.22% of the total amount of carbon in *E. coli* cells). Moreover nanoSIMS imaging reveals that such incorporation of exogenous carbon is homogeneous in all the cells of the same type. Consequently, isotopic composition of single cells could be determined precisely with SIMSISH, provided that nanoSIMS is calibrated with a reference. However, fixation and hybridization influence on isotopic composition may not be exactly the same for other types of cells (e.g. with other membrane types), and will have to be evaluated in the course of further studies.

### 2. Double Labeling of Complex Sample

**Figure 3:**
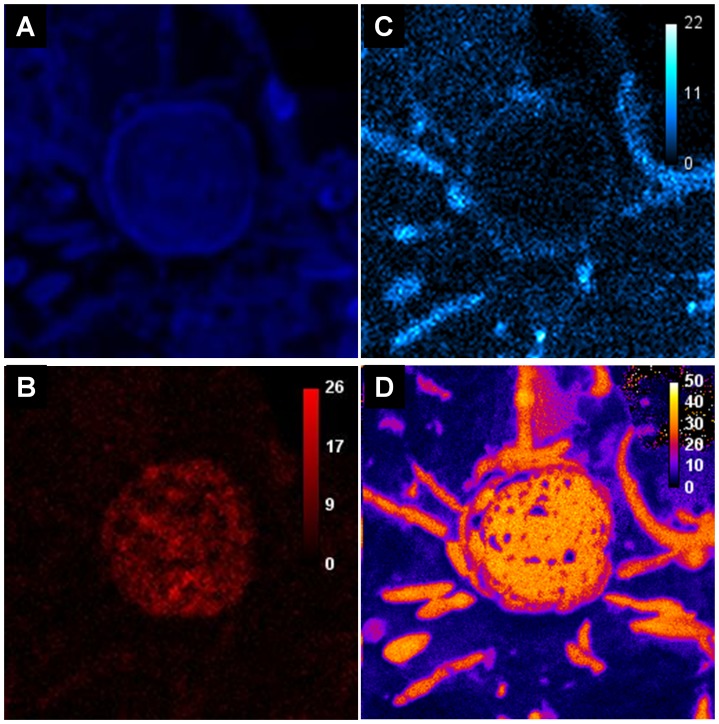
NanoSIMS images obtained from a double-labelled complex sample (^13^C-labeled methanol degrading anaerobic digester). NanoSIMS images obtained from acomplex sample(^13^C-labeled methanol degrading anaerobic digester) labelled with a generalist bacterial iodinated probe (EUBI) and a Methanosarcina genera specific brominated probe (MS1414). Panel (a) shows the secondary ion of ^32^S^−^ image as an image of total biomass. Panel (b) shows the secondary ion of ^81^Br^−^ image as an indication of archaeal cell identity. Panel (c) shows the secondary ion of ^127^I^−^ image as an indication of total bacteria. Panels (d) shows the ^13^C Isotopic abundance map.

A ^13^C isotopic enrichment measurement of cells from a complex sample (^13^C-labeled methanol degrading anaerobic batch digester) is given to illustrate the potential of the SIMSIH technique. A double labeling SIMSISH experiment enabled to both measure the isotopic enrichment of a archaeal cluster affiliated to Genus *Methanosarcina* (MS1414 probe labeled with iodine) and of the nearby bacterial cells (EUB338 probe labeled with bromine). A set of images showing sulfur emission, bromine emission, iodine emission, and ^13^C abundance map is presented on Figure 3 a, b, c and d. Clear bromine and iodine signals, corresponding to probes hybridization, were observed and enabled the identification of both *Methanosarcina* archaea and bacterial cells at the same time. This is, to our knowledge, the first successful attempt of FISH double labeling observation with nanoSIMS on a complex sample. Precise ^13^C labeling enrichment was realized at a smaller observation scale. Figure 3b shows a set of images corresponding to ^12^C and ^13^C ions emission, as well as ^13^C % in cells. The mean ^13^C enrichment of archaeal cluster was 30.0% (standard error = 1.3%), and the mean enrichment of bacteria was 24.4% (standard error = 2.5%), showing that both types of microorganisms were involved in methanol degradation at different levels. These values will be used in degradation models in further studies to unravel the function of each microorganism in the degradation process.

An accurate isotopic ratio measurement associated with phylogenetic identification will open the door to advanced understanding of metabolic networks and microbial interactions in complex ecosystems. Moreover additional phylogenetic groups could be targeted simultaneously by using more probes.

## Conclusion

We have demonstrated that SIMSISH sample preparation procedure has very limited influence on the isotopic composition of *E. coli* cells. Therefore, by giving an accurate measurement of 13C fluxes within single cells, this approach can provide information on metabolic activity at single cell level and thus offers insights into the distribution of microbial activities in and among individual cells of probe-identified populations. Multiple labelling seems to be a very attractive prospect to perform several isotopic measurements and identification at the same time or to study specific syntrophic interactions. Combined with stable isotope probing [14], SIMSISH could be an elegant tool to decipher networks of biogeochemical processes catalysed by uncultured microorganisms within complex environments. Indeed, the possibility to follow accurately the fluxes of stable isotope at a particular time point opens the possibility of implanting the measurements into degradation models to resolve specific function directly in complex ecosystems.

## Supporting Information

Figure S1 **Figure S1 Fluorescent image of hybridized E. coli cells with a generalist bacterial iodinated probe (EUBI).** Fluorescent image of hybridized E. coli cells (80% of ^13^C enrichment group) with a generalist bacterial iodinated probe (EUBI). Fluorescent signal is clear and enables to visualize hybridized cells.
(TIF)

Figure S2 **Figure S2: NanoSIMS images obtained from hybridized E. coli cells with a generalist bacterial iodinated probe (EUBI).** NanoSIMS images obtained from hybridized E. coli cells (80% of ^13^C enrichment group) with a generalist bacterial iodinated probe (EUBI). Panel (a) shows the secondary ion of ^32^S^−^ image as an image of total biomass. Panel (b) shows the secondary ion of ^127^I^−^ image as an indication of hybridized cells. 88% of cells have a clear iodine hybridization signal.
(TIF)

Figure S3 **Figure S3^13^C isotopic abundance map of mixed 10%, 20%, 40%, 80% ^13^C enriched E. coli cells hybridized with a generalist bacterial iodinated probe (EUBI).** NanoSIMS observation showing ^13^C isotopic abundance map of mixed 10%, 20%, 40%, 80% ^13^C enriched E. coli cells hybridized with a generalist bacterial iodinated probe (EUBI). Lateral resolution of nanoSIMS provides an efficient discrimination between lateral neighbor cells. Demarcation between the different types of cells is clear and level of enrichment inside cells is very regular and not affected by the surrounding cells.
(TIF)

File S1 **Estimation of the influence of the addition of probes during hybridization step of SIMSISH on the isotopic composition of cells based on calculation and detailed values of isotopic measurements.**
(DOCX)
