# Supplementary figures and images for: SIMSISH Technique Does Not Alter the Apparent Isotopic Composition of Bacterial Cells
Source: PLoS One. 2013 Oct 29;8(10):e77522. doi: 10.1371/journal.pone.0077522 (PMC3812282; doi:10.1371/journal.pone.0077522)

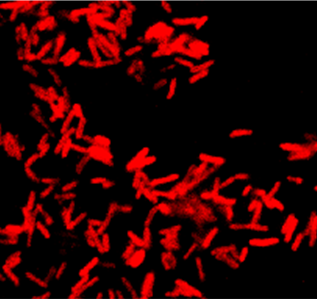

Supplement: Figure S1 — Fluorescent image of hybridized E. coli cells with a generalist bacterial iodinated probe (EUBI). Fluorescent image of hybridized E. coli cells (80% of 13C enrichment group) with a generalist bacterial iodinated probe (EUBI). Fluorescent signal is clear and enables to visualize hybridized cells. (TIF) [file pone.0077522.s001.tif]

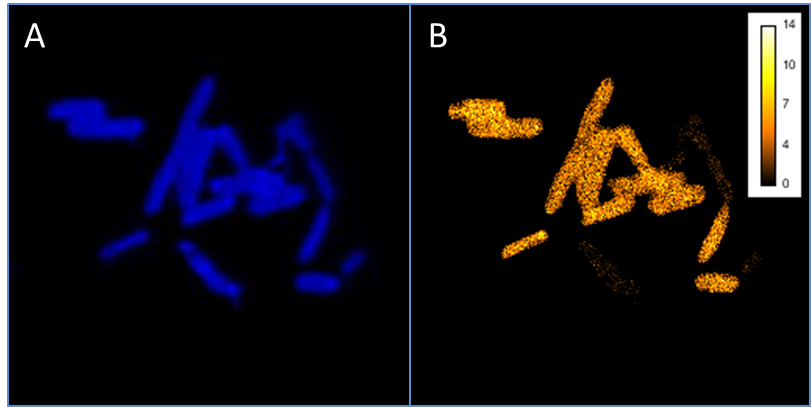

Supplement: Figure S2 — NanoSIMS images obtained from hybridized E. coli cells with a generalist bacterial iodinated probe (EUBI). NanoSIMS images obtained from hybridized E. coli cells (80% of 13C enrichment group) with a generalist bacterial iodinated probe (EUBI). Panel (a) shows the secondary ion of 32S− image as an image of total biomass. Panel (b) shows the secondary ion of 127I− image as an indication of hybridized cells. 88% of cells have a clear iodine hybridization signal. (TIF) [file pone.0077522.s002.tif]

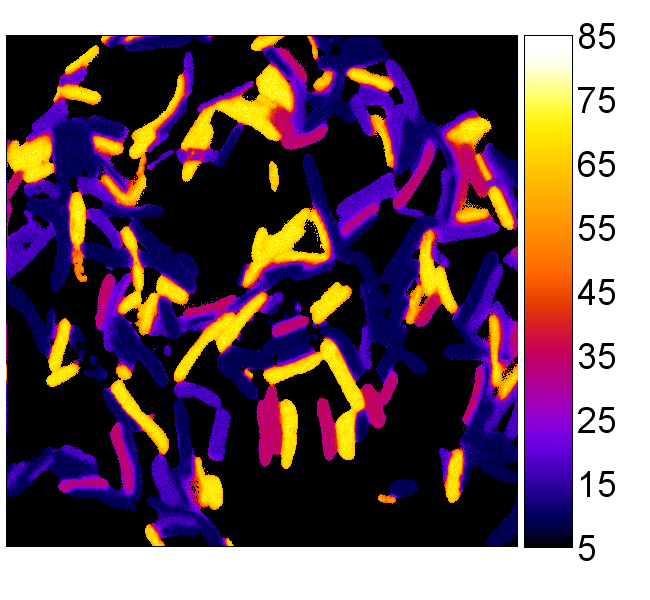

Supplement: Figure S3 — 13C isotopic abundance map of mixed 10%, 20%, 40%, 80% 13C enriched E. coli cells hybridized with a generalist bacterial iodinated probe (EUBI). NanoSIMS observation showing 13C isotopic abundance map of mixed 10%, 20%, 40%, 80% 13C enriched E. coli cells hybridized with a generalist bacterial iodinated probe (EUBI). Lateral resolution of nanoSIMS provides an efficient discrimination between lateral neighbor cells. Demarcation between the different types of cells is clear and level of enrichment inside cells is very regular and not affected by the surrounding cells. (TIF) [file pone.0077522.s003.tif]
