# Supplementary material for: SIMSISH Technique Does Not Alter the Apparent Isotopic Composition of Bacterial Cells
Source: PLoS One. 2013 Oct 29;8(10):e77522. doi: 10.1371/journal.pone.0077522 (PMC3812282; doi:10.1371/journal.pone.0077522)
Supplement: File S1 — Estimation of the influence of the addition of probes during hybridization step of SIMSISH on the isotopic composition of cells based on calculation and detailed values of isotopic measurements. (DOCX) [file pone.0077522.s004.docx]

**SIMSISH technique does not alter the apparent isotopic composition of bacterial cells**

**Authors:**

**Olivier Chapleur^a^, Ting-Di Wu^b,c^, Jean-Luc Guerquin-Kern^b,c^, Laurent Mazeas^a^, Théodore Bouchez^a^**

**Affiliations:**

1. *Irstea, UR HBAN, 1 rue Pierre-Gilles de Gennes - CS 10030, F-92761 Antony Cedex, France*
2. *INSERM, U.759, Orsay 91405, France*
3. *Institut Curie, Laboratoire de Microscopie Ionique, Orsay 91405, France*

**Corresponding author:**

email [olivier.chapleur@irstea.fr](mailto:olivier.chapleur@irstea.fr); Tel. +33140966506; Fax +33140966199

*Irstea, UR HBAN, 1 rue Pierre-Gilles de Gennes - CS 10030, F-92761 Antony Cedex, France*

**Supporting information:**

1. **Estimation of the influence of the addition of probes during hybridization step of SIMSISH on the isotopic composition of cells based on calculation**

A quick calculation was made to estimate the influence of addition of probes on the isotopic composition of cells during hybridization. An *E. coli* cell contains between 6800 and 72000 ribosomes [[1](#_ENREF_1)]. An *E. coli* cell contains 2.8*10^^-13^ g of dry matter [[2](#_ENREF_2)], composed of 47% of carbon [[3](#_ENREF_3)]. Therefore each *E. coli* cell contains 1.3*10^^-13^ g of carbon. Each of the probes we used contains 6 G, 4 T, 2 A and 6 C bases, and one Cy3 dye, *i.e.* 203 atoms of carbon (6*10+4*10+2*10+6*9+29=203). Considering the highest possible number of ribosomes, if all ribosomes were hybridized, up to 14.6*10^^6^ atoms of carbon would be introduced in cells with probes (203*72000), i.e. 2.9*10^^-16^ g of carbon. The maximum amount of carbon introduced with probes during the hybridization would be 0.22 % of the total amount of carbon contained in cells. This theoretical estimation shows that the addition of probes during SIMSISH procedure should not significantly affect the isotopic composition of cells measured with nanoSIMS.

**References**

1. Bremer H, PP D (1996) Modulation of chemical composition and other parameters of the cell by growth rate. In: Neidhardt FC, editor Escherichia coli and Salmonella ASM Press; Washington DC: 1553–1569.

2. Neidhardt FC, H.E. U (1996) Chemical Composition of Escherichia coli in Escherichia coli and Salmonella: Cellular and Molecular Biology. Vol 1., Chapter 3. ASM Press

3. Heldal M, Norland S, Tumyr O (1985) X-ray microanalytic method for measurement of dry matter and elemental content of individual bacteria. Applied and Environmental Microbiology 50: 1251-1257.

**Tables:**

***Table A.*** *Isotopic composition of E.coli cells measured with EA-IRMS, detailed values*

| Sample | Percentage of ^13^C in culture media | | | | | | | | | |
| --- | --- | --- | --- | --- | --- | --- | --- | --- | --- | --- |
|  | 1.10% | 2.08% | 3.06% | 6.00% | 10.89% | 20.68% | 40.26% | 59.84% | 79.42% | 99.00% |
| Untreated *E.coli* cells, assay 1 | 1.10% | 2.01% | 2.91% | 5.69% | 10.31% | 19.70% | 38.55% | 57.70% | 77.78% | 97.47% |
| Untreated *E.coli* cells, assay 2 | 1.12% | 2.00% | 2.92% | 5.69% | 10.31% | 19.72% | 38.52% | 57.70% | 77.85% | 91.11% |
| Fixed *E.coli* cells, assay 1 | 1.10% | 1.98% | 2.87% | 5.54% | 10.05% | 19.10% | 37.32% | 56.48% | 76.27% | 96.00% |
| Fixed *E.coli* cells, assay 2 | 1.10% | 1.95% | 2.80% | 5.63% | 10.17% | 19.27% | 37.74% | 56.66% | 76.64% | 95.15% |
| Fixed and hybridized *E.coli* cells, assay 1 | 1.10% | 1.99% | 2.88% | 5.59% | 10.11% | 19.39% | 38.01% | 57.24% | 77.24% | 96.63% |

***Legend****: Isotopic composition of untreated, fixed, fixed/hybridized E.coli cells measured with EA-IRMS on dried cells pellets, detailed values*

***Table B.*** *Isotopic composition of individual untreated E.coli cells measured with nanoSIMS, detailed values*

| Percentage of ^13^C in culture media | | | |
| --- | --- | --- | --- |
| 0.11 | 0.21 | 0.40 | 0.79 |
| 9.58 | 18.20 | 36.56 | 70.02 |
| 9.55 | 17.99 | 36.40 | 71.08 |
| 9.88 | 18.10 | 36.77 | 70.72 |
| 9.71 | 18.58 | 37.17 | 71.11 |
| 9.75 | 18.52 | 35.66 | 71.38 |
| 9.54 | 17.88 | 35.65 | 70.89 |
| 9.93 | 18.73 | 37.19 | 70.66 |
| 9.50 | 17.95 | 35.91 | 70.16 |
| 9.35 | 18.76 | 35.86 | 69.21 |
| 9.81 | 17.94 | 36.45 | 70.07 |
| 9.38 | 17.95 | 36.00 | 70.36 |
| 9.33 | 18.11 | 35.83 | 71.42 |
| 9.27 | 18.86 | 35.84 | 70.86 |
| 9.24 | 18.98 | 36.49 | 71.02 |
| 9.36 | 18.44 | 35.78 | 71.59 |
| 9.31 | 18.64 | 37.14 | 71.05 |
| 9.99 | 18.21 | 35.68 | 70.85 |
| 9.45 | 18.53 | 36.88 | 70.69 |
| 9.61 | 18.72 | 35.88 | 71.24 |
| 9.33 | 17.93 | 35.92 | 70.75 |
| 9.34 | 17.82 | 35.95 | 71.15 |
| 9.49 | 17.97 | 35.90 | 70.71 |
| 9.44 | 17.90 | 37.90 | 70.54 |
| 9.33 | 17.77 | 36.26 | 69.76 |
| 9.37 | 17.83 | 35.92 | 69.73 |
| 9.41 | 17.80 | 36.30 | 70.24 |
| 8.94 | 17.93 | 36.11 | 70.26 |
| 9.39 | 17.75 | 35.95 | 70.33 |
| 9.28 | 17.59 | 35.40 | 72.35 |
|  | 18.13 | 35.64 | 72.42 |
|  | 17.95 | 36.01 | 72.41 |
|  | 17.84 | 35.73 | 72.46 |
|  | 17.84 | 35.48 | 71.81 |
|  | 17.77 | 35.43 | 71.53 |
|  | 18.80 | 36.13 | 73.13 |
|  | 18.04 | 35.31 | 72.39 |
|  | 18.52 | 35.48 | 72.80 |
|  | 17.75 | 36.65 | 72.08 |
|  | 18.81 | 35.49 | 72.42 |
|  | 17.80 | 35.67 | 72.89 |
|  | 18.07 | 36.57 | 72.23 |
|  | 17.92 | 36.66 | 72.76 |
|  | 18.01 | 35.51 | 70.49 |
|  | 17.81 | 35.72 | 72.18 |
|  | 17.86 | 35.54 | 72.49 |
|  | 17.72 | 35.90 | 72.11 |
|  | 18.00 | 35.74 | 70.70 |
|  | 17.95 | 35.86 | 72.21 |
|  | 17.96 | 35.68 | 72.39 |
|  | 17.68 |  | 72.04 |
|  | 17.98 |  | 71.80 |
|  | 17.84 |  | 71.99 |
|  | 18.12 |  | 72.29 |
|  | 18.76 |  | 71.75 |
|  | 17.73 |  | 70.58 |
|  | 17.99 |  | 71.63 |
|  | 18.17 |  |  |
|  | 17.95 |  |  |
|  | 18.04 |  |  |
|  | 18.07 |  |  |
|  | 18.78 |  |  |

***Legend****: Isotopic composition of individual untreated E.coli cells measured with nanoSIMS, detailed values*

***Table C.*** *Isotopic composition of individual hybridized E.coli cells measured with nanoSIMS, detailed values*

| Percentage of ^13^C in culture media | | | |
| --- | --- | --- | --- |
| 0.11 | 0.21 | 0.40 | 0.79 |
| 9.60 | 17.14 | 34.68 | 70.51 |
| 9.72 | 17.13 | 35.07 | 69.32 |
| 9.46 | 17.58 | 35.07 | 70.80 |
| 9.35 | 17.67 | 35.41 | 70.89 |
| 9.51 | 17.85 | 35.12 | 70.80 |
| 9.38 | 17.70 | 35.40 | 70.72 |
| 9.46 | 17.76 | 35.40 | 71.32 |
| 9.20 | 17.76 | 35.05 | 70.31 |
| 9.63 | 17.84 | 35.32 | 70.05 |
| 9.34 | 17.64 | 35.09 | 70.10 |
| 9.58 | 17.83 | 34.73 | 70.24 |
| 9.12 | 17.69 | 35.43 | 69.32 |
| 9.23 | 17.80 | 35.30 | 70.48 |
| 9.56 | 18.04 | 35.34 | 70.32 |
| 9.40 | 17.66 | 35.61 | 70.09 |
| 8.98 | 17.73 | 35.75 | 69.91 |
| 9.56 | 17.62 | 34.53 | 70.26 |
| 9.18 | 17.51 | 35.73 | 70.53 |
| 9.31 | 17.61 | 35.62 | 71.01 |
| 9.39 | 17.68 | 34.64 | 70.35 |
| 9.29 | 17.51 | 34.85 | 70.91 |
| 9.41 | 17.44 | 35.06 | 70.01 |
| 9.44 | 17.19 | 35.13 | 70.88 |
| 9.19 | 17.79 | 35.05 | 70.64 |
| 9.42 | 17.49 | 35.27 | 70.98 |
| 9.26 | 17.96 | 35.18 | 70.03 |
| 8.93 | 17.83 | 34.86 | 70.41 |
| 9.22 | 17.86 | 35.02 | 70.50 |
| 9.36 | 17.84 | 35.32 | 70.29 |
| 9.31 | 17.59 | 35.40 | 70.75 |
| 9.30 | 17.73 | 34.98 | 70.32 |
| 9.11 | 17.77 | 34.64 | 69.43 |
|  | 17.86 | 35.27 | 70.39 |
|  | 17.49 |  | 70.09 |
|  | 17.72 |  | 68.90 |
|  | 18.06 |  | 69.94 |
|  | 17.66 |  | 71.37 |
|  | 17.81 |  | 70.69 |
|  | 17.97 |  | 71.83 |
|  | 17.05 |  | 72.43 |
|  | 17.68 |  | 71.57 |
|  |  |  | 72.34 |
|  |  |  | 72.23 |
|  |  |  | 72.25 |
|  |  |  | 71.25 |
|  |  |  | 71.90 |
|  |  |  | 72.65 |
|  |  |  | 72.17 |
|  |  |  | 72.20 |
|  |  |  | 71.95 |
|  |  |  | 72.09 |
|  |  |  | 72.31 |
|  |  |  | 72.61 |
|  |  |  | 71.95 |
|  |  |  | 71.94 |
|  |  |  | 71.67 |
|  |  |  | 72.01 |
|  |  |  | 71.05 |
|  |  |  | 71.56 |
|  |  |  | 70.95 |
|  |  |  | 72.23 |
|  |  |  | 71.87 |

***Legend****: Isotopic composition of individual hybridized E.coli cells measured with nanoSIMS, detailed values*
